# Smoking duration before first childbirth: an emerging risk factor for breast cancer? Results from 302,865 Norwegian women

**DOI:** 10.1007/s10552-013-0213-1

**Published:** 2013-05-01

**Authors:** Eivind Bjerkaas, Ranjan Parajuli, Elisabete Weiderpass, Anders Engeland, Gertraud Maskarinec, Randi Selmer, Inger Torhild Gram

**Affiliations:** 1 Department of Community Medicine, Faculty of Health Sciences, University of Tromsø, Tromsø, Norway; 2 Department of Medical Epidemiology and Biostatistics, Karolinska Institutet, Stockholm, Sweden; 3 Department of Genetic Epidemiology, Folkhälsan Research Center, Samfundet Folkhälsan, Helsinki, Finland; 4 Department of Research, Cancer Registry of Norway, Oslo, Norway; 5 Division of Epidemiology, Department of Pharmacoepidemiology, Norwegian Institute of Public Health, Oslo, Norway; 6 Department of Public Health and Primary Health Care, University of Bergen, Bergen, Norway; 7 Cancer Center, University of Hawaii, Honolulu, HI USA; 8 Norwegian Centre for Integrated Care and Telemedicine, University Hospital of North Norway, Tromsø, Norway

**Keywords:** Breast cancer, Smoking, Childbirth, Cohort study, CONOR

## Abstract

**Purpose:**

Recently, The International Agency for Research on Cancer classified cigarette smoking as possibly carcinogenic to the human breast. Since some new cohort studies have suggested that this risk is confined to women who started to smoke before first childbirth, we wanted to examine the association between smoking and breast cancer, with a focus on time of smoking initiation in relation to the first childbirth.

**Methods:**

We followed 302,865 Norwegian women born between 1899 and 1975, recruited from 1974 to 2003, by linkage to national registries through December 2007. We used Cox proportional hazard models to estimate hazard ratios (HR) and 95 % confidence intervals (CI).

**Results:**

During more than 4.1 million person-years of follow-up, we ascertained 7,490 cases of primary invasive breast cancer. Compared with never smokers, ever smokers had a 15 % (HR = 1.15, 95 % CI 1.10–1.21) increased risk of breast cancer overall and also a significantly increased risk of breast cancer in the three most exposed categories of age at smoking initiation (parous women), number of cigarettes smoked per day, years of smoking duration and number of pack-years. Ever smokers who started to smoke more than 1 year after the first childbirth had not an increased risk (HR = 0.93, 95 % CI 0.86–1.02), while those who initiated smoking more than 10 years before their first childbirth had a 60 % (HR = 1.60, 95 % CI 1.42–1.80) increased risk of breast cancer, compared with never smokers.

**Conclusion:**

Smoking initiation before the first childbirth increases the risk of breast cancer.

## Introduction

Breast cancer is by far the most frequently diagnosed cancer among women worldwide, representing 23 % of all female cancers in 2008 [1]. Established risk factors for breast cancer include age, having none or few children, late age at first childbirth, early menarche, late age at menopause, being postmenopausal, obesity and alcohol consumption [2, 3].

In 2009, the Canadian Expert Panel on tobacco smoke and breast cancer risk summarized in their report that there was evidence for an increased risk of breast cancer for those smoking many years and pack-years of cigarettes, compared with never smokers [2]. Recently, the International Agency for Research on Cancer classified cigarette smoking as possibly carcinogenic to the human breast [4].

In Norway, breast cancer among women comprised more than 20 % of all female cancer cases with 2,839 women diagnosed with the disease in 2010. Long-term observations from the Norwegian Cancer Registry show that there has been a genuine increase in risk of breast, lung and colorectal cancer among women from 1956 to 2005 [5]. During these 50 years, the prevalence for daily smoking for women has changed considerably. The prevalence was 23 % in 1954, the peak was at 37 % in 1970 and then, the prevalence of daily smokers stabilized at around 32 % for the rest of the century [6]. Globally, if smoking causes even a small increase in risk, this will account for a substantial number of new cases since breast cancer is such a common disease.

Some cohort studies [7–13] have suggested that the increased risk of breast cancer associated with smoking is confined to women who started to smoke before their first childbirth. The purpose of the study was to examine the association between smoking and breast cancer, with a focus on time of smoking initiation in relation to the first childbirth.

## Materials and methods

### Study population

The study population comprised 302,865 Norwegian women born between 1899 and 1975, recruited into three large Norwegian prospective cohort studies conducted by the National Health Screening Service (now the Norwegian Institute of Public Health): the Norwegian Counties Study (1974–1988), the 40 years Cohort (1985–1999) and the Cohort of Norway (CONOR, 1994–2003).

The protocols for the above described surveys were similar. Selection of participants in the studies was usually based on year of birth and residence (municipality or county). All surveys had a baseline questionnaire, which included detailed assessments of smoking habits, physical activity and other lifestyle factors. As a part of a short health examination at the screening facility, body height and weight were measured in a standardized way by a trained nurse, which allowed us to calculate body mass index (BMI, kg/m^2^). In most surveys, the attendees were given another questionnaire to be completed at home and mailed back in a prestamped envelope. The wording of the questionnaires was standardized from 1994 onwards, when the CONOR collaboration was initiated [14–16]. From 1994, all subjects recruited gave written informed consent to participate in the studies, while before 1994, returning the completed questionnaire was considered sufficient as acceptance to participate in the studies. The response rate in the studies varied from around 56 to 88 % [17]. The present study was approved by the Regional Committee for Medical Research Ethics South-East, Norway.

### Exposure information

The smoking questions were similar, but not identical, across all surveys. The questionnaires asked about current and former daily smoking habits, smoking duration, average number of cigarettes smoked per day, and former smokers were asked about time since quitting. Only the CONOR study asked about age at smoking initiation. In the other surveys, we calculated this variable for both current (age at enrollment minus duration of smoking in years) and former (age at enrollment minus years since quitting and duration of smoking) smokers. We collapsed current and former smokers to ever smokers. We further categorized ever smokers according to the following factors: age at smoking initiation (≤16, 17–19, 20–24, ≥25 years), numbers of cigarettes smoked per day (1–9, 10–19, ≥20), smoking duration in years (1–19, 20–29, 30–39, ≥40) and number of pack-years (i.e., number of cigarettes smoked per day, divided by 20, multiplied by the duration of smoking in years; 0–9, 10–19, ≥20) .

For parous women, the variable “years of smoking before first childbirth” was calculated as age at first childbirth minus age at smoking initiation. This variable was further classified according to time of smoking initiation in relation to first childbirth: [more than 1 year after, around (i.e., 1 year before to 1 year after), 1–3 years before, 4–6 years before, 7–10 years before, ≥11 years before first childbirth] Women, who had been smoking for ≤20 years or for >20 years, were classified as short- and long-term smokers, respectively. All women not being current or former smokers were classified as never smokers. They constitute the reference group throughout the paper unless otherwise noted.

The subjects were categorized into three groups based on the level of physical activity at time of enrollment: sedentary (reading, watching television and sedentary activity), moderate (walking, bicycling, and/or similar activities ≥4 h per week) and heavy (light sports or heavy gardening ≥4 h per week, heavy exercise or daily competitive sports). The most recent information regarding duration of education obtained from Statistics Norway was used to assign subjects to one of three categories according to years of education: <10, 10–12, ≥13.

Information on number of children and age at first childbirth was also obtained through linkages to Statistics Norway.

### Follow-up and endpoints

We followed all participants through linkages to the Cancer Registry of Norway and the Central Population Register using the unique 11-digit personal identification number to identify all cancer cases, emigrations and deaths, respectively. The national registries are both accurate and virtually complete [18]. The start of follow-up was set to January 1 the year after completing the baseline questionnaire. Person-years were calculated from the start of follow-up to the date of breast cancer diagnosis, the date of any other incident cancer diagnosis (except basal cell carcinoma), emigration, death or the end of follow-up (31 December 2007), whichever occurred first. Breast cancer cases were classified according to the Seventh Revision of the International Classification of Diseases (code 170). All prevalent cancer cases (*n* = 7,138), women without information on smoking status (*n* = 2,808), education level (*n* = 6,913), BMI (*n* = 2,478) and physical activity (*n* = 4,207), were excluded, leaving 302,865 women in the analytical cohort.

### Statistical analysis

We used the *t* test for examining differences in the distribution of selected characteristics among parous ever smokers according to time of smoking initiation in relation to first childbirth (before or after).The Cox proportional hazards model was used with age as the underlying time scale to estimate multivariate-adjusted hazard ratios (HRs) with 95 % confidence intervals (CIs) for the associations between different measures of smoking exposure [age at smoking initiation for nulliparous and parous women (<15, 15–19, 20–24, ≥25 years), numbers of cigarettes smoked per day (≤5, 6–10, 11–15, ≥16), smoking duration in years (<11, 11–20, 21-30, ≥31), number of pack-years (≤5, 6–10, 11–15, ≥16) and for parous smokers smoking initiation in relation to first childbirth [more than 1 year after, around (i.e., 1 year before to 1 year after), 1–3 years before, 4–6 years before, 7–10 years before, ≥11 years before] and breast cancer with never smokers as the reference group. We repeated the analyses for smoking initiation in relation to first childbirth restricting the analyses to parous ever smokers, using women who initiated smoking more than 1 year after first childbirth as the reference group. These analyses were further stratified according to years of smoking duration (≤20, >20) and number of cigarettes (≤10, >10).

Entry time was defined as age at enrollment, and exit time was age at diagnosis of breast cancer, the date of any incident cancer diagnosis (except basal cell carcinoma), emigration, death or the end of follow-up (31 December 2007), whichever occurred first.

The co-variates included in the final models, decided a priori, were age at enrollment (continuous variable), years of education (<10, 10–12, ≥13), number of children (0, 1–2, ≥3), age at first childbirth (<20, 20–24, 25–29, ≥30 years), BMI (<25, 25–29, ≥30 kg/m^2^) and physical activity (sedentary, moderate, heavy). We analyzed the age and multivariate adjusted HR’s with 95 % CI for breast cancer risk according to the selected co-variates included in the multivariate analyses.

We did sensitivity analyses among women who were asked about and had reported on alcohol consumption (*n* = 114,804). Alcohol consumption was categorized as; less than weekly, weekly or more often than weekly. We analyzed the age and multivariate adjusted HR’s with 95 % CI for breast cancer risk according to alcohol consumption. Data shown in Table 3. We analyzed age and multivariate adjusted HR’s with 95 % CI for breast cancer risk among ever smokers adjusting for alcohol consumption. The increased HR estimates for ever compared with never smokers were no longer significant for this analysis including 1,759 cases. The magnitude of these HR estimates did not differ materially from those presented in the paper (Data not shown).

It was not possible to calculate age at smoking initiation due to missing information for 50 % of parous former smokers (*n* = 33,391). Thus, the analyses on smoking before first childbirth are based on 127,757 women, including 2,966 cases of breast cancer. We performed analysis with 50 years as a proxy for age at menopause and stratified analysis on smoking exposure according to attained age less than 50 years old and 50 years and older. This procedure is described in detail elsewhere [19]. The results were considered significant if the *p* value was <0.05. All *p* values are two-sided. The analyses were done in STATA, version 12.0 (StataCorp, College Station, TX, USA).

## Results

During more than 4.1 million person-years of observation, 7,490 histologically confirmed cases of primary invasive breast cancer were identified during a median follow-up time of 14 years. Table 1 shows the selected characteristics of the study population stratified by birth cohort and smoking status. The mean age at first childbirth was around 24 years for the four cohorts displayed. From the oldest (women born before 1946) to the youngest (women born after 1955) cohort, the mean age at smoking initiation decreased, and the proportion of women who started to smoke before their first childbirth increased steadily (Table 1).

**Table 1:** Selected characteristics of the study population, stratified by birth cohort and smoking status, among 302,865 Norwegian women (1974–2003)

Birth cohort (year of birth)	<1946		1946–1950		1951–1955		>1955		Total
Characteristics/smoking status	Ever	Never	Ever	Never	Ever	Never	Ever	Never	
Subjects	34,950	35,088	49,165	30,067	57,766	32,997	37,481	25,351	302,865
Age^a^, (mean), SD	50 ± 12	54 ± 14	42 ± 3	42 ± 3	42 ± 3	42 ± 3	39 ± 5	38 ± 6	44 ± 9
Person years of follow-up	657,363	654,555	781,699	480,232	665,721	381,025	325,583	218,137	4,164,314
Number of primary invasive breast cancers	1,226	1,136	1,620	927	1,232	713	384	252	7,490
Follow-up years, (median)	19	19	16	16	12	12	9	9	14
≥13 years of education (%)	10	14	14	29	18	38	16	35	21
Age at diagnosis, (mean), SD	61 ± 9	63 ± 11	52 ± 5	52 ± 5	49 ± 4	49 ± 5	46 ± 4	46 ± 4	54 ± 9
Nulliparous women	4,456	5,112	4,496	3,210	6,272	3,738	4,929	4,310	36,523
Parous women	30,494	29,976	44,669	26,857	51,494	29,259	32,552	21,041	266,342
Number of children, (mean), SD	2 ± 2	2 ± 2	2 ± 1	2 ± 1	2 ± 1	2 ± 1	2 ± 1	2 ± 1	2 ± 1
Age at first childbirth, (mean), SD	24 ± 4	25 ± 5	23 ± 4	24 ± 4	23 ± 5	25 ± 5	24 ± 5	26 ± 5	24 ± 5
Age at smoking initiation^b^ (year), SD	24 ± 8	NA	21 ± 5	NA	19 ± 5	NA	17 ± 3	NA	20 ± 6
Age at smoking initiation among nulliparous^b^ (year), SD	24 ± 7	NA	21 ± 5	NA	19 ± 4	NA	18 ± 4	NA	20 ± 6
Women smoking before first childbirth^b^ (%)	62	NA	63	NA	81	NA	94	NA	76
Body mass index^a^ (kg/m²)	25	26	24	24	24	25	25	25	25
Level of physical activity, heavy^a,c^ (%)	11	12	10	12	31	33	27	32	21
Smoking status^a^ (%)	50	50	62	38	64	36	60	40	*59*

Ever smoker: current or former daily smokers

*SD* standard deviation,* NA* not applicable

^a^At enrollment, ^b^Information not available for all smokers, ^c^Light sports or heavy gardening ≥4 h per week, heavy exercise or daily competitive sports

Table 2 presents parous never and ever smokers, the latter group also stratified by timing of smoking initiation in relation to first childbirth. Compared with women who started to smoke after, those who started before their first childbirth were younger at enrollment, at diagnosis, at age of smoking initiation were older at first childbirth and had on average smoked more years and more cigarettes per day (Table 2).

**Table 2:** Selected characteristics for parous ever and never smokers, according to start of smoking before, around or after first childbirth

	Parous ever smokers				Parous never smokers
	Timing of smoking initiation^a^				
Characteristics	Before first childbirth^b^	Around first childbirth^c^	After first childbirth^d^	*p* value^e^	NA
Number of subjects	85,922	18,574	23,261		107,133
Age, SD	43 ± 7	43 ± 6	45 ± 8	<0.001	44 ± 10
Total years of follow-up	1,017,860	274,265	362,485		1,516,653
No of primary invasive breast cancers	1,892	485	589		2,578
Follow-up (years)	12	15	16		14
Age at diagnosis, SD	52 ± 7	53 ± 8	55 ± 9	<0.001	54 ± 10
Age at first childbirth, SD	24 ± 5	21 ± 3	21 ± 3	<0.001	25 ± 5
Age at smoking initiation, SD	18 ± 3	21 ± 3	28 ± 7	<0.001	NA
Smoking duration (years), SD^a^	19 ± 8	20 ± 6	15 ± 7	<0.001	NA
Number of cigarettes pr day, SD^a^	12 ± 6	12 ± 5	10 ± 5	<0.001	NA
Number of pack years, SD^a^	12 ± 8	12 ± 7	8 ± 6	<0.001	NA

Numbers given as mean unless specified

Analysis on 127,757 women and 2,966 cases with information on smoking initiation, and all parous never smokers

*SD* standard deviation,* NA* not applicable

^a^Smoking status given at enrollment

^b^>1 year before first childbirth

^c^≤1 year before and ≤1 year after first childbirth

^d^>1 year after first childbirth

^e^ *T* test between women initiating smoking before and after first childbirth when applicable

Table 3 shows, after multivariate adjustments, a positive association for level of education and alcohol consumption and risk of breast cancer and an inverse association between breast cancer risk and number of children, early age at first childbirth, BMI and physical activity (Table 3).

**Table 3:** Age and multivariate adjusted hazard ratios (HR) and 95 % confidence intervals (CI) for breast cancer according to selected covariates among 302,685 Norwegian women (1974–2003)

	Person years	Cases	Age adjusted	Multivariate adjusted^a^
*Duration of education (years)*				
<10	1,158,622	1,864	Ref.	Ref.
10–12	2,231,711	3,987	1.27 (1.20–1.34)	1.28 (1.20–1.35)
≥13	773,981	1,639	1.53 (1.42–1.63)	1.54 (1.43–1.65)
Sum	4,164,314	7,490		
*Number of children*				
0	478,233	959	Ref.	Ref.
1–2	2,080,768	4,068	0.99 (0.93–1.07)	0.87 (0.78–0.98)
≥3	1,605,313	2,463	0.74 (0.69–0.80)	0.66 (0.59–0.74)
Sum	4,164,314	7,490		
Age at first childbirth (year)*				
<20	502,441	792	Ref.	Ref.
20–24	1,779,522	2,903	0.99 (0.92–1.07)	0.92 (0.85–1.00)
25–29	994,875	1,898	1.16 (1.07–1.26)	0.99 (0.91–1.08)
≥30	409,214	938	1.39 (1.26–1.52)	1.09 (0.98–1.20)
Sum	3,686,052*	6,531*		
*Body mass index (kg/m²)* ^*b*^				
<25	2,681,616	4,894	Ref.	Ref.
25–29	1,098,495	1,965	0.92 (0.88–0.97)	0.96 (0.91–1.00)
≥30	384,203	631	0.82 (0.76–0.90)	0.88 (0.81–0.96)
Sum	4,164,314	7,490		
*Physical activity* ^*b,c*^				
Sedentary	885,181	1,633	Ref.	Ref.
Moderate	2,544,962	4,614	1.01 (0.96–1.07)	0.97 (0.92–1.03)
Heavy	734,171	1,243	1.03 (0.96–1.11)	0.97 (0.90–0.99)
Sum	4,164,314	7,490		
Alcohol consumption**				
<Weekly	703,988	1,154	Ref.	Ref.
Weekly	152,877	310	1.20 (1.06–1.37)	1.18 (1.04–1.34)
>Weekly	127,106	295	1.32 (1.16–1.50)	1.26 (1.10–1.44)
Sum	983,971**	1,759**		

* Nulliparous (*n* = 36,523) not included

** Only women with alcohol information included (*n* = 114,804)

^a^Adjusted for age, education level, number of children, age at first childbirth, BMI, age at enrollment and physical activity when applicable

^b^At enrollment

^c^Physical activity: sedentary (reading, watching television and other sedentary activity), moderate (walking, bicycling or similar activities ≥4 h per week), heavy (light sports or heavy gardening ≥4 h per week, heavy exercise or daily competitive sports)

Table 4 shows that the multivariate HR estimate was similar for current (HR = 1.14, 95 % CI 1.08–1.20) and former (HR = 1.17, 95 % CI 1.10–1.24) smokers, compared with never smokers. Ever smokers had a 15 % (HR = 1.15, 95 % CI 1.10–1.21) increased risk of breast cancer, and they also had a significantly increased risk of breast cancer in the three most exposed categories of age at smoking initiation (parous women), number of cigarettes smoked per day, years of smoking duration and number of pack-years, compared with never smokers. Ever smokers who started to smoke more than one year after the first childbirth did not have an increased risk of breast cancer, compared with never smokers (HR = 0.93, 95 % CI 0.86–1.02). Ever smokers who had initiated smoking more than ten years before their first child birth had a 60 % (HR = 1.60, 95 % CI 1.42–1.80) increased risk of breast cancer, compared with never smokers. Excluding never smokers, a trend test across the categories for smoking exposure displayed in the table, all yielded significant *p* values (all *p* trend’s <0.001). Compared with never smokers, ever smokers had a 15 % (HR = 1.15, 95 % CI 1.09–1.22) and a 12 % (HR = 1.12, 95 % CI 0.98–1.26) increased risk of pre- and postmenopausal breast cancer, respectively.

**Table 4:** Multivariate adjusted hazard ratios (HR) and 95 % CIs for breast cancer according to various measures of smoking exposures at enrollment, with never smokers as the reference group, for 302,865 Norwegian women (1974–2003)

	Subjects	Cases	Multivariate adjusted HR (95 % CI)^a^
*Smoking exposures*			
Smoking status			
Never	123,503	3,028	Ref.
Former	64,021	1,581	1.17 (1.10–1.24)
Current	115,341	2,881	1.14 (1.08–1.20)
Sum	302,865	7,490	
Ever	179,362	4,462	1.15 (1.10–1.21)
*Ever smokers*			
Age at smoking initiation for nulliparous women (years)			
≥25	2,788	91	1.01 (0.80–1.26)
20–24	5,001	140	1.03 (0.85–1.25)
15–19	8,087	153	1.01 (0.83–1.22)
<15	960	16	1.33 (0.81–2.21)
Sum	16,836	400	
		*p* trend*	<0.001
Age at smoking initiation for parous women (years)			
≥25	20,362	540	0.99 (0.90–1.09)
20–24	35,822	987	1.20 (1.11–1.29)
15–19	65,131	1,348	1.33 (1.24–1.42)
<15	6,442	88	1.30 (1.05–1.61)
Sum	127,757	2,963	
		*p* trend*	<0.001
Smoking duration (years)^b^			
<11	48,446	1,167	1.08 (1.02−1.15)
11–20	70,384	1,822	1.12 (1.05−1.35)
21–30	53,420	1,313	1.26 (1.18−1.35)
≥31	5,379	131	1.31 (1.09−1.57)
Sum	177,629	4,433	
		*p* trend*	<0.001
Number of cigarettes smoked per day			
≤5	32,591	786	1.08 (0.99–1.17)
6–10	77,405	1,844	1.09 (1.03–1.16)
11–15	41,266	1,087	1.26 (1.17–1.35)
≥16	26,567	725	1.30 (1.20–1.41)
Sum	177,829	4,442	
		*p* trend*	<0.001
Number of pack-years^c^			
≤5	56,837	1,360	1.07 (1.00–1.14)
6–10	46,041	1,150	1.11 (1.04–1.19)
11–15	35,149	889	1.18 (1.09–1.27)
≥16	35,152	942	1.34 (1.25–1.45)
Sum	173,179	4,341	
		*p* trend*	<0.001
Smoking initiation in relation to first childbirth for parous women (years)			
After first childbirth (>1 year)	23,261	589	0.93 (0.86–1.02)
Around childbirth^d^	18,574	482	1.09 (0.99–1.20)
1–3	21,29	524	1.21 (1.10–1.33)
4–6	28,367	588	1.14 (1.03–1.24)
7–10	21,256	450	1.34 (1.21–1.48)
≥11	14,970	330	1.60 (1.42–1.80)
Sum	127,757	2,963	
		*p* trend**	<0.001

* Trend test between four levels of smoking categories excluding never smokers

** Trend test between five levels of smoking categories excluding never smokers and those smoking >1 year after first childbirth

^a^Adjusted for age, education level, number of children, age at first childbirth, BMI, age at enrollment and physical activity when applicable

^b^Total number of years smoked

^c^Pack years: Number of cigarettes smoked per day multiplied by number of years smoked, divided by 20. One pack has 20 cigarettes

^d^1 year before to 1 year after first childbirth

**Fig. 1:**
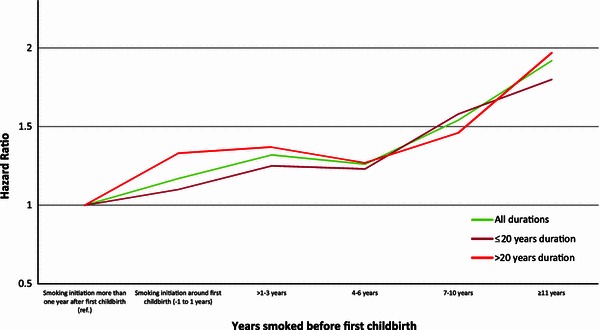
Multivariate adjusted HR of breast cancer risk according to time of smoking initiation among ever smokers with smoking initiation after first childbirth as reference (HR = 1.0), for all smoking durations, for ≤20 years of duration, for >20 years duration, among 302,865 Norwegian women (1974–2003)

**Fig. 2:**
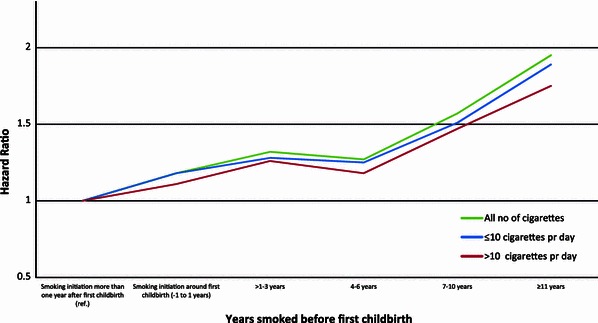
Multivariate adjusted HR of breast cancer risk according to time of smoking initiation among ever smokers with smoking initiation after first childbirth as reference (HR = 1.0), for all number of cigarettes, for ≤10 cigarettes per day, for >10 cigarettes per day, among 302,865 Norwegian women (1974–2003)

Figures 1 and 2 display the HR’s of breast cancer for parous ever smokers, with women who initiated smoking more than 1 year after first childbirth as the reference group, for the different categories of smoking initiation in relation to first childbirth overall and stratified by years of smoking [≤20, >20 (Fig. 1)] and overall and stratified by number of cigarettes [≤10, >10 (Fig. 2)]. When compared with those who initiated smoking more than 1 year after first childbirth, the figures show that for all six categories, the risk of breast cancer increases with number of years smoked before first childbirth (Figs. 1, 2).

## Discussion

Our study finds that female ever smokers have an increased risk of breast cancer compared with never smokers. This increased risk does not vary according to menopausal status. A causal interpretation of our results is supported by the presence of a consistent dose–response association between the various measures of smoking exposure (i.e., age at smoking initiation, smoking duration in years, average number of cigarettes smoked per day and number of pack-years) and risk of breast cancer. We do not find any increased risk of breast cancer for women who started to smoke after their first childbirth. Furthermore, we find a consistent dose–response relationship between the number of years smoked before the first childbirth and the risk of breast cancer for both short- and long-term smokers, as well as for women smoking ten or less or more than ten cigarettes daily.

Since 2004, more than eight large prospective cohort studies including more than 500 cases of breast cancer have been published on the relationship between smoking and breast cancer risk [7–13, 20]. Compared with never smokers, five [9–11, 13, 20] studies reported an increased risk among current and three [8, 13, 20] studies reported an increased risk for former smokers. Our risk estimates are of the same magnitude as the corresponding risk estimates in these studies.

Cohort studies reporting on the association between smoking duration and breast cancer risk found an increased breast cancer risk [7–11, 20], which was significant in four [7, 8, 11, 20] out of the six studies for the highest category of years of smoking duration. Compared with never smokers, the highest reported increased risk of breast cancer of 50 % was found for women who had been smoking for 40 years or more [11]. Our study finds risk estimates of a similar magnitude only for women who have been smoking for 10 years or more before their first childbirth.

A meta-analysis from 2011, including eight cohort and 15 case–control studies published between 1988 and 2010, examined the association between smoking before first childbirth and the risk of breast cancer [21]. The analyses revealed a 10 % increase in risk of breast cancer for women who initiated smoking before first childbirth compared with never smokers. Nevertheless, DeRoo et al. concluded that a causal association between smoking and breast cancer was unlikely. In contradiction to this, the Canadian Expert Panel summarized in their report that smoking before first full-term pregnancy could be associated with a causally increased risk of breast cancer [2].

Since 2004, seven prospective cohort studies have reported that women smoking before first childbirth have a small, but increased risk of breast cancer of the same magnitude as we do [7–13]. In our previous study, [7] we found, as we do in the present study, that women who initiated smoking after first childbirth did not have an increased breast cancer risk compared with never smokers.

The second report on smoking and breast cancer from the Nurses’ Health Study, including more than 1,000 cases of breast cancer, showed an increased risk of breast cancer of 42 % for women who had smoked from 15 to 19 years before their first childbirth [8]. Our results, which for this analysis were based on almost three times as many breast cancer cases, find an increased risk for all categories of women who initiated smoking more than 1 year before first childbirth. The third and most recent report on smoking and breast cancer from the Nurses’ Health Study reported an increased risk of 18 % with a significant test for trend for every increase of 20 pack-years smoked before first childbirth compared with never smokers [13]. This study, including close to 9,000 breast cancer cases, found that women in the most exposed category (smoking >16 pack-years before first childbirth) had a 25 % increased risk of breast cancer compared with never smokers.

We find that there was a large increase in proportion of women who started to smoke before the first child birth from the oldest to the youngest birth cohorts. This difference between older and younger birth cohorts may explain why older studies including older birth cohort did not find any association between smoking and breast cancer risk [22–25], while the more recent with younger birth cohorts do [7–13].

Our study has several major strengths. The study is based on a large prospective cohort population from Norway comprising women who have been followed for many years from all 19 counties, with virtually complete follow-up. We have a high proportion female ever smokers and were able to examine the association with smoking initiation in relation to first childbirth in detail. The long follow-up period resulting in a large number of cases, gives us more stable risk estimates and results that are less prone to chance. We were able to stratify all the analyses according to different measures of smoking exposure, and also, the smoking histories were obtained at enrollment and, hence, are not subject to recall bias. Another strength is that we focused our analyses on the comparison between ever smokers and never smokers. Thus, it is only never smokers who could possibly change smoking status during follow-up. Since very few Norwegians start to smoke after the age of 30 and the mean age at enrollment for our study is 44 years, we are confident that the possible changes in smoking status among the never smokers during follow-up did not influence our risk estimates. We had information on, and were able to control for, established risk factors for breast cancer, many of which varied according to smoking status. Our analyses of the covariates showed the expected dose–response relationship with breast cancer risk, which substantiates the validity of our data. Furthermore, we do find the expected positive association between different measures of smoking exposure and risk of colon cancer for the ever smokers in this cohort [26]. Breast cancer screening was not yet common in Norway during the first 20 years of follow-up in our study. However, the Norwegian Breast Cancer Screening Programme began screening women aged 50–69 at the end of 1995 and gradually expanded to become national by 2005 [5]. Since this implementation of screening became nationwide just 2 years before the end of our follow-up period, we find it unlikely that this should have biased our results to a great extent.

Our study has several limitations. We consider our lack of ability to adjust for several of the established breast cancer risk factors (e.g., age at menarche, use of oral contraceptives, age at menopause, use of postmenopausal hormone therapy and alcohol consumption), due to lack of question about this in the questionnaires, as the most important. However, our sensitivity analysis including women with information on alcohol consumption (*n* = 114,804) who all were enrolled after 1995 did not change our risk estimates materially although we lost 76 % of the total follow-up time and 77 % of the cases. That our results do not change when we adjust for alcohol consumption is in accordance with our previous study [7] from Norway and the California Teachers Study cohort [10] which both found an increased risk of breast cancer also for non-drinkers who were smoking compared with those who were not. Another limitation is that we lack information about hormone receptor status and other tumor characteristics.

Several studies have found a small increased risk of breast cancer associated with passive smoking [2, 20, 27, 28]. We were not able to exclude passive smokers from the reference group. Also, 10 % of the female Norwegian population has reported to be occasional smokers during the last four decades [29]. As our questionnaires asked about daily smoking, occasional smokers most likely have answered “no” and thereby been categorized as never smokers. This “pollution” of the reference group may have biased our results toward the null.

Already in 1982, Russo et al. hypothesized that the mammary tissue is more susceptible to carcinogenic exposures between menarche, when the cells are undifferentiated, and the last trimester of the first pregnancy when the evolution make the cells differentiated [30, 31]. Cigarette smoke contains more than twenty substances that have been identified to induce mammary tumors in rodents, and these chemicals are also found in human breast tissue [32]. Our results are in support of Russo’s hypothesis. However, given the lack of information on several well-known breast cancer risk factors, our results must be evaluated with caution.

The findings from our large cohort study add more strength to the concept that also breast cancer is a tobacco related cancer. Our results also support the notion that smoking for many years before the first childbirth especially increases the risk of breast cancer. Globally, female teenagers and young adults who smoke should be informed that quitting smoking today, not in 10 years, is of great importance when it comes to their risk of breast cancer, the most common female cancer.
